# Caveolin-1 Controls Vesicular TLR2 Expression, p38 Signaling and T Cell Suppression in BCG Infected Murine Monocytic Myeloid-Derived Suppressor Cells

**DOI:** 10.3389/fimmu.2019.02826

**Published:** 2019-12-03

**Authors:** Vini John, Leigh A. Kotze, Eliana Ribechini, Gerhard Walzl, Nelita Du Plessis, Manfred B. Lutz

**Affiliations:** ^1^Institute for Virology and Immunobiology, University of Würzburg, Würzburg, Germany; ^2^Division of Molecular Biology and Human Genetics, Faculty of Medicine and Health Sciences, DST-NRF Centre of Excellence for Biomedical Tuberculosis Research, South African Medical Research Council Centre for Tuberculosis Research, Stellenbosch University, Cape Town, South Africa

**Keywords:** myeloid-derived suppressor cell (MDSC), caveolin-1 (Cav-1), TLR2, TLR4, BCG, T cell suppression

## Abstract

Monocytic myeloid-derived suppressor cells (M-MDSCs) and granulocytic MDSCs (G-MDSCs) have been found to be massively induced in TB patients as well in murine Mtb infection models. However, the interaction of mycobacteria with MDSCs and its role in TB infection is not well studied. Here, we investigated the role of Cav-1 for MDSCs infected with *Mycobacterium bovis* Bacille-Calmette-Guerín (BCG). MDSCs that were generated from murine bone marrow (MDSCs) of wild-type (WT) or *Cav1*^−/−^ mice upregulated Cav-1, TLR4 and TLR2 expression after BCG infection on the cell surface. However, Cav-1 deficiency resulted in a selective defect of intracellular TLR2 levels predominantly in the M-MDSC subset. Further analysis indicated no difference in the phagocytosis of BCG by M-MDSCs from WT and *Cav1*^−/−^ mice or caveosome formation, but a reduced capacity to up-regulate surface markers, to secrete various cytokines, to induce iNOS and NO production required for suppression of T cell proliferation, whereas Arg-1 was not affected. Among the signaling pathways affected by Cav-1 deficiency, we found lower phosphorylation of the p38 mitogen-activated protein kinase (MAPK). Together, our findings implicate that (i) Cav-1 is dispensable for the internalization of BCG, (ii) vesicular TLR2 signaling in M-MDSCs is a major signaling pathway induced by BCG, (iii) vesicular TLR2 signals are controlled by Cav-1, (iv) vesicular TLR2/Cav-1 signaling is required for T cell suppressor functions.

## Introduction

Tuberculosis (TB) is an airborne infectious disease caused by the intracellular pathogen *Mycobacterium tuberculosis* (Mtb) which is transmitted by aerosol route. The WHO reports that 10.4 million people suffered from active TB and 1.3 million died of it in 2017 (1). Bacillus Calmette-Guerin (BCG) is the only vaccine available against TB. Despite its widespread use in new-born babies, BCG does not prevent adult pulmonary disease satisfactorily and therefore, has not reduced the global TB burden. The reasons for the varying efficacy of BCG in protection against pulmonary TB are not completely understood.

Myeloid-derived suppressor cells (MDSCs) are major immuno-regulatory cells. MDSCs consists of granulocytic MDSCs (G-MDSCs) and monocytic MDSCs (M-MDSCs). G-MDSCs and M-MDSCs have relatively low phagocytic activity compared to dendritic cells and macrophages but they have increased levels of reactive oxygen species (ROS), NO production, arginase-1(Arg-1) expression, PGE_2_ and a number of anti-inflammatory cytokines (2). In mice, G-MDSCs can be identified best as CD11b^+^ Ly-6G^+^ Ly-6C^low^ and M-MDSCs as CD11b^+^ Ly-6G^−^ Ly-6C^hi^ (3), although these markers are not specific.

We found that MDSCs were expanded in the blood of TB patients and decreased after successful chemotherapy (4), and that vaccinations using Mtb can accumulate MDSCs in the spleens of mice (5). In a murine model of TB infection, MDSCs phagocytosed Mtb and secreted IL-10, IL-6, and IL-1α (6). A higher frequency of MDSCs was associated with higher levels of IL-4α and targeted depletion of MDSCs by anti-Gr-1 antibodies or all-trans-retinoic acid (ATRA) resulted in a better outcome of the disease (6). Accumulation of MDSCs in the lung and blood of TB patients correlated with enhanced L-arginine catabolism and NO production (7). Both monocytic and granulocytic subsets were accumulated at the infection site as well as in the blood depending on the severity of disease and other factors (4, 7).

Several reports suggest the adverse effects of MDSCs on anti-TB immunity for T cell proliferation and activation (4, 6–8). Therefore, MDSCs could be considered as cellular targets for host-directed therapies against active TB disease, but this requires a better understanding of mycobacteria interaction with MDSCs. Here, we used G-MDSCs and M-MDSCs that were generated from murine bone marrow (MDSCs) following a protocol we published earlier (9). This allowed us to study MDSC interaction with mycobacteria in more detail.

Mycobacterial ligands are recognized by defined pattern recognition receptors such as TLR2 and TLR4 to induce immune responses by macrophages and dendritic cells (10). Although MDSCs also express TLRs, their activation induces immunosuppressive responses, a phenomenon that can be exploited for microbial immune evasion (11). TLR2 activation by specific agonists increase the potential of MDSCs to suppress anti-tumor immune responses (12). Similarly, TLR4 activation through LPS has been shown to be essential for MDSC expansion, activation, and suppression (13). Several TLRs can interact with plasma membrane components such as Cav-1 to control phagocytosis and cell activation. Cav-1 is a structural protein component in lipid raft invaginations of the plasma membrane which regulates lipid metabolism, signal transduction, and membrane trafficking. Immune cells such as dendritic cells, macrophages, monocytes, neutrophils, B cells are known to express Cav-1 (14–17). Depending on the cell type and pathogen stimulus, Cav-1 can have different functions. In endothelial cells, Cav-1 interacts with TLR4 for NF-κB activation resulting in the secretion of pro-inflammatory cytokines (18). Mutational studies have shown that Cav-1 binding to TLR4 is required for suppression of cytokine production (19). Other reports have shown that Cav-1 regulates TLR4 signaling in murine peritoneal macrophages (14). In a murine chronic asthma model, inhibition of airway inflammation occurred via Cav-1 through TLR2 mediated activation of MyD88 and NF-κB (20). Cav-1 is found in the bulb-shaped pits of the plasma membrane and are involved in the internalization of pathogens such as SV40 virus (21), echovirus (22), respiratory syncitia virus (23), *S. typhimurium*, and certain FimH-expressing bacteria (24). Caveosome formation by Cav-1 association to phagosomes has been proposed to serve as an intracellular niche for pathogen survival by forming caveosomes (25). However, there are conflicting reports questioning the existence of caveosomes (26). From these findings, we hypothesized that Cav-1 may play an important role in MDSCs for mycobacterial uptake and activation.

Caveolae in lipid-raft microdomains are associated with cell signaling cascades by directly interacting with several proteins such as Src family tyrosine kinases, endothelial NO synthase (eNOS) and the insulin receptor (27). During bacterial infections, Cav-1 has a multi-faced role. On one hand, *Cav1*^−/−^ mice displayed higher bacterial burdens, decreased phagocytosis ability, higher production of inflammatory cytokines and increased mortality in *Pseudomonas aeruginosa* and *Salmonella typhimurium* infection (28, 29). On the other hand, *Cav1^−/−^* mice showed decreased mortality and low levels of inflammation mediated by eNOS derived NO (30). However, the role of Cav-1 in mycobacterial infections and their role in MDSCs have not been investigated.

In this study we found upregulation of surface Cav-1, TLR2, and TLR4 expression in both G-MDSCs and M-MDSCs subsets of MDSCs after BCG infection. Using murine MDSCs from WT and *Cav1*^−/−^ mice, we found that Cav-1 does not play a role in BCG phagocytosis or caveosome formation but rather influences MDSC activation through intracellular TLR2 but not TLR4 signaling via p38 MAPK supporting NO production to suppress T cell proliferation. This study provides insights into the functional role of Cav-1 for TLR2 signals after mycobacterial infections in MDSCs.

## Materials and Methods

### Animals and Ethics Statement

C57BL/6 and *Cav1*^−/−^ mice (B6. Cg-Cav^tm1Mls^/J, JAX mice) were bred under specific pathogen-free conditions in our animal facility at the Institute of Virology and Immunobiology at Würzburg, Germany and were used at an age of 6–10 weeks. The *in vitro* experiments with BM or other murine organs from mice were performed according to the German animal Protection Law (TSchG) and under control of the local authorities (Regierung von Unterfranken).

### BCG

BCG-GFP (31) was cultured in Middlebrook 7H9 broth medium (BD Difco) having 0.05% tween-80, 0.05% glycerol and 10% albumin-dextrose-catalase (ADC) supplement. Log-phase cultures were harvested by centrifugation at 1,000 rpm for 10 min. Bacterial aggregates were removed by additional centrifugation at 50 rpm for 10 min. Bacillary count was determined on basis of optical density at 600 nm. BCG was grown at 35° in the presence of 30 μg/ml kanamycin (Sigma).

### Reagents

LPS (100 ng/ml) and Pam_3_CSK_4_ (1 μg/ml) were purchased from Sigma-Aldrich. Pharmacological inhibitors cytochalasin D (1 μg/ml), filipin III (3 μg/ml), simvastatin (50 nM, β-cyclodextrine (1 mM) were purchased from Sigma-Aldrich.

### Murine Bone Marrow-Derived Myeloid Derived Suppressor Cells (MDSCs)

MDSCs were generated as previously described (9). Briefly, tibiae or femurs were removed from 4 to 10-week-old mice. BM was flushed out with a PBS-filled sterile 10 ml syringe. BM cells was washed once by centrifugation by 1,000 rpm for 10 min. BM cells were then cultured in complete RPMI medium supplemented with 10% GM-CSF for 3 days. On day 3, non-adherent and semi-adherent cells were harvested and washed in complete RPMI medium prior to *in vitro* stimulation assays.

### Pharmacological Inhibition for BCG Uptake

WT MDSCs were incubated at 1.5 × 10^6^/well in a 24-well plate with cytochalasin-D (1 μg/ml), filipin III (1 μg/ml), simvastatin (50 nM), or β-cyclodextrine (1 mM) for 1 h and then stimulated with BCG-GFP at MOI of 2, 5, or 10 for 6 h. Cells were then analyzed by flow cytometry for BCG uptake by GFP detection in G-MDSC and M-MDSC subsets.

### *In vitro* Stimulation of MDSCs With BCG

MDSCs from WT or *Cav1*^−/−^ were added in a 24-well-plate (1.5 × 10^6^ cells per well). BCG was added to cultures at indicated multiplicities of infection (MOI). Cells were harvested after 16 h and analyzed for the surface expression of TLR2, TLR4, Cav-1, PDL-1, E-Cadherin, CD40, CD69 or intracellular expression of TLR2, TLR4, iNOS, and arginase1. To analyze the production of various cytokines and nitric oxide, MDSCs were stimulated with BCG at 2,5,10 MOI and culture supernatants were collected after 16 h.

### Antibodies

#### For Flow Cytometry

Ly-6C (clone: HK1.4), Ly-6G (clone: 1A8), CD11b (clone: M1/70), rabbit caveolin1 (#3238,CST), PDL1 (clone: 10F.9G2), TLR4 (clone: MTS510), TLR2 (clone: 6C2), iNOS (clone: CXNFT), Arginase 1 (clone: A1exF5), CD4 (clone: GK1.5) CD8 (clone: 53-6.7), CD40 (clone: 3/23), CD69 (clone: H1.2F3), phospho-p38 MAPK (clone: 4NIT4KK), phospho-AKT (clone: SDRNR). All antibodies were directly fluorochrome conjugated and purchased from BioLegend, except unconjugated Cav-1 which was purchased from Cell Signaling Technologies and detected with donkey anti-rabbit-DyLight^TM^ 649 secondary antibody (Jackson Immuno Research).

#### For Western Blot

All antibodies were purchased from Cell Signaling Technologies except for α-Tubulin which was purchased from Santa Cruz (#sc-8035). Rabbit phospho-p38 MAP Kinase (#9211), rabbit p38 MAPK (#9212), rabbit phospho-AKT (#4060), rabbit AKT (#4691), and secondary antibody HRP (horseradish peroxidase)-anti-rabbit (#7074) was used.

#### For Microscopy

Rabbit caveolin1 (#3238, CST), TLR2-Alexa 647 (clone: 6C2), TLR4-Biotin (clone: MTS510), DAPI (eBioscience). Following labeled secondary reagents were used: donkey anti-rabbit-DyLight^TM^ 649 (Jackson Immuno Research), Cy3-Streptavidin (BioLegend).

### Flow Cytometry

For surface staining of *in vitro* stimulated MDSCs, 1.5 × 10^6^ cells were harvested from the 24 well plates and washed with FACS buffer (1x PBS supplemented with 0.1% BSA and 0.1% NaN_3_). Cells were then re-suspended in 100 μl FACS Buffer and stained with antibodies against surface molecules for 20 min on ice before flow cytometry.

For Intracellular staining 1.5 × 10^6^ cells were fixed with 2% formaldehyde for 20 min at room temperature after surface staining as described before and then washed in FACS buffer. Cells were then stained intracellularly for 45 min with 100 μl antibodies diluted in 1x intracellular staining perm wash buffer (BioLegend) at room temperature. Cell were then resuspended in 100 μl FACS buffer before flow cytometry.

For phosphorylated stainings of p-AKT and p-38 markers 1.5 × 10^6^ cells were fixed with 2% formaldehyde for 20 min at room temperature after surface staining described before. Cells were then incubated in “IC fixation buffer” (eBioscience) for 30 min at room temperature. After washing with FACS buffer, cells were incubated in ice-cold methanol. Cells were then washed twice with FACS buffer and then incubated with 100 μl respective phospho-markers diluted in FACS buffer for 60 min at room temperature. Cell were resuspended in FACS buffer before flow cytometry.

MDSCs were analyzed for Annexin-V (BD Pharmingen) staining, following the instructions provided by the manufacturer. In brief, 1.5 × 10^6^ cells were washed with FACS buffer before incubating with surface antibodies (Ly-6G, Ly-6C, and CD11b) diluted in FACS buffer for 20 min. Cell death was analyzed by staining the cells for 20 min with a staining mix composed of 1x Annexin-V binding buffer containing annexin-V (BD Pharmingen).

For all stainings 50,000 events were acquired on a BD LSRII with DIVA software (BD Biosciences, San Jose, USA). Data analysis was done on FlowJo software (Tree Star, USA).

### MDSC-T Cell Suppressor Assay

MDSCs from WT or *Cav1*^−/−^ mice were pre-activated at 1.5 × 10^6^/well in a 24-well plate with BCG for 1 h. T cells from Spleen and lymph nodes of syngeneic mouse were then labeled with the proliferation dye Cell Trace Violet (Thermo Fisher Scientific) and 2 × 10^6^ cells were added into a 48-well flat bottom plate (Greiner). T cells were stimulated with soluble anti-CD3(1 μg/ml) and anti-CD28 (1 μg/ml). BCG-activated MDSCs were then added at 1:10 (2 × 10^5^ cells/well) or 1:30 (6.6 × 10^4^ cells/well) ratios. Co-cultures were analyzed after 3 days. Cells were harvested and stained for CD4 and CD8 and analyzed by flow cytometry to detect T cell suppression. Proliferated T cells dilute the tracer during cell division and so can be measured as % Cell Trace Violet-low expressing cells.

### Cytokine Estimation With ELISA

Cytokine (IL-6, IL-10, IL-12p40, TNF-α, and IL-1β) levels in MDSCs culture supernatants 1.5 × 10^6^/well in a 24-well plate were determined using ELISA kits purchased from BioLegend, San Diego, CA, according to the instructions provided by the manufacturer.

### NO Measurement

NO production was determined by measuring its stable end product nitrite, using the standard Griess reagent. Briefly, 50 μl of supernatants from 1.5 × 10^6^/well in a 24-well plate were added to 96 well plate, followed by 50 μl of 1:1 ratio mixed Griess reagents A = 0.1% sulphanilamide and B = 0.1% N-1-napthylethylenediamine dihydrochloride (NED). Absorbance at 492 nm was measured by microplate reader and standard curves were created based on the NaNO_2_ optical density (OD) readings. From this standard curve, sample concentrations were calculated.

### Cytospins and Immunofluroscence Staining

MDSCs from WT and *Cav1*^−/−^ mice were stimulated at 1.5 × 10^6^/well in a 24-well plate with BCG-GFP for 16 h. Cells were then centrifuged onto a glass slide by cytospin at 600 g for 10 min. Cells on the slides were fixed with 4% paraformaldehyde (PFA)/PBS 20 min. After washing with PBS, cells were permeabilized with 0.1% Triton X-100 for 5 min and then blocked with 5% BSA for 30 min. Cells with primary antibody diluted in 1% BSA were incubated overnight at 4°C. Next Day, corresponding secondary antibody was added for 1 h at room temperature. Nuclei were stained using DAPI (eBioscience). Slides mounted with Fluoromount-G (SouthernBiotech) were analyzed by confocal laser-scanning microscope (LSM 780, Zeiss).

### Western Blot

Cellular lysates (1.5 × 10^6^) were lysed at 4°C for 1 h in 1 ml of lysis buffer consisting of 50 mM Tris-HCl [pH 8.0], 150 mM sodium chloride [NaCl], 1.0% Igepal CA-630 [NP-40], 0.5% sodium deoxycholate, 0.1% sodium dodecyl sulfate [SDS] containing complete protease inhibitor cocktail (Sigma) and 1 mM dl-dithiothreitol (DTT). The protein quantification was done using the bicinchoninic acid (BCA) assay. Equal amounts of proteins were heated at 95°C for 5 min in reducing Laemmli buffer (50 mM Tris HCl [pH 6.8], 2% SDS, 10% glycerol, 1% β-mercaptoethanol, 12.5 mM EDTA, 0.02% bromophenol blue) and subjected to 10% SDS-PAGE. Proteins were blotted semidry on nitrocellulose membranes (Applichem), followed with 5% BSA or 5% milk in PBS with 0.05% Tween 20. Then membranes were incubated with specific primary antibodies overnight followed by HRP-conjugated secondary antibodies. Signals were visualized with the help of chemiluminescent FemtoMax supersensitive HRP substrate (Rockland). Quantification of protein bands was done using Li-cor software. The fold changes in phosphorylated proteins were normalized to the band densities of total protein and/or Total Revert stain (Li-cor). Western blotting was repeated 3–5 times, and representative images are shown.

### Normalization of FACS Data

Normalization of FACS surface staining data was performed using the mean fluorescence intensities (MFI) values of individual experiments for the expression of Cav-1, TLR2, and TLR4. Unstimulated WT-MDSCs were considered as 1 and fold change was calculated accordingly. Normalization of phosphorylated markers such as p38 and pAKT was done in comparison to WT-unstimulated control that were considered as 1 and for all at 0 min.

### Statistical Analysis

Statistical analyses were performed with help of GraphPad Prism 6.0 (Graphpad, USA) or Fiji ImageJ for co-localization studies and calculating Pearson's coefficients (National Institute of Health, USA). Data from the experiments are presented as mean values ± standard error of the mean (SEM) or standard deviation (SD) as indicated, and the statistical tests are indicated in the legends.

## Results

### Cav-1 Is Upregulated Upon BCG Infection but Its Deficiency Does Not Affect TLR4 or TLR2 Surface Expression on Murine MDSCs

**Figure 1:**
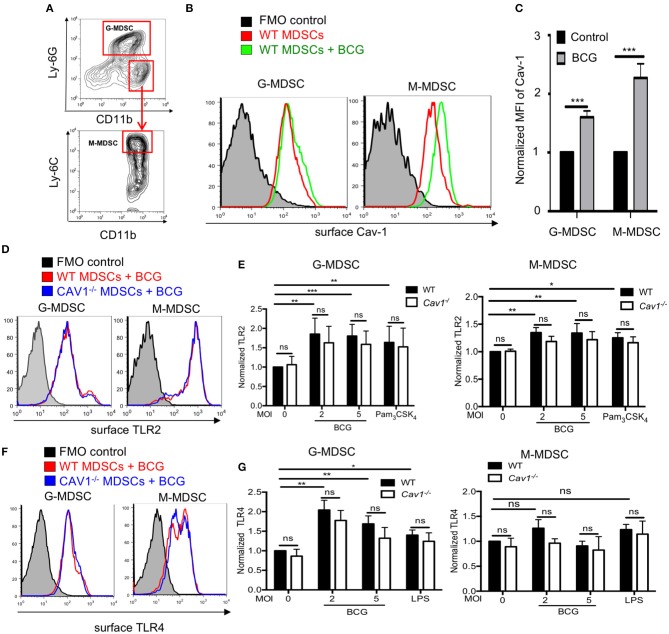
Upregulation of TLR2 and TLR4 on MDSCs after BCG infection is independent of Cav-1 expression. **(A)** MDSCs were differentiated from WT mice by culturing them *in vitro* with GM-CSF for 3 days and then analyzed by flow cytometry. Gating strategy to define G-MDSCs and M-MDSCs after FSC/SSC live gating and doublet exclusion by expression of CD11b, Ly-6C, and Ly-6G. **(B)** MDSCs from WT mice were stimulated with BCG at 5 MOI or left unstimulated and analyzed after 16 h for surface Cav-1 expression on G-MDSCs and M-MDSCs. **(C)** Pooled and normalized data of several experiments as performed for **(B)**. **(D,F)** MDSCs from WT or *Cav1*^−/−^ mice were stimulated for 16 h with BCG and analyzed for surface TLR2 or TLR4 expression by flow cytometry. **(E)** Pooled and normalized data of several experiments as performed for **(D)** and additional stimulation at different MOIs or the TLR2 agonist Pam_3_CSK_4_. **(G)** Pooled and normalized data of several experiments as performed for **(F)** and additional stimulation at different MOIs or the TLR4 agonist LPS. Normalization of **(C,E,G)** was done according to unstimulated WT control. Data represent for **(C)** *n* = 7, for **(E)** *n* = 7, and for **(G)** *n* = 6 independent experiments. ***P < 0.001; **P < 0.01; *P < 0.05; ns, not significant by unpaired, two-tailed, student's *t*-test.

Cav-1 has been demonstrated to be upregulated in macrophages upon HIV infection (32). We investigated the functional role of Cav-1 during mycobacterial infections by using defined MDSCs generated from BM cells of WT or *Cav1*^−/−^ mice. Murine G-MDSCs and M-MDSCs were identified by their differential expression of CD11b, Ly-6G, and Ly-6C (Figure 1A). Of note, MDSCs could be generated from both WT and *Cav1*^−/−^ mice, and BCG infection did not induce cell death of MDSCs (Supplementary Figure 1). Both subsets of MDSCs up-regulated Cav-1 expression on the cell surface upon BCG infection on WT MDSCs (Figures 1B,C). Similarly, the up-regulation of both surface TLR2 and TLR4 was observed with different MOIs or after exposure to their respective ligands for TLR2 (Pam_3_CSK_4_) or TLR4 (LPS) detected by flow cytometry (Figures 1D–G), except that there was only a trend for up-regulation of surface TLR4 expression between unstimulated or BCG infected M-MDSCs (Figure 1G). For TLR2 and TLR4 no significant differences between WT and *Cav1*^−/−^ MDSCs could be observed (Figures 1B–G). Thus, our data indicate that although Cav-1 is increased in murine G-MDSC and M-MDSC upon BCG infection, its genetic deficiency does not alter the surface expression of TLR2 and TLR4.

### Cav-1 Deficient BCG-Stimulated MDSCs Have Reduced Intracellular Levels of TLR2 but Not TLR4

**Figure 2:**
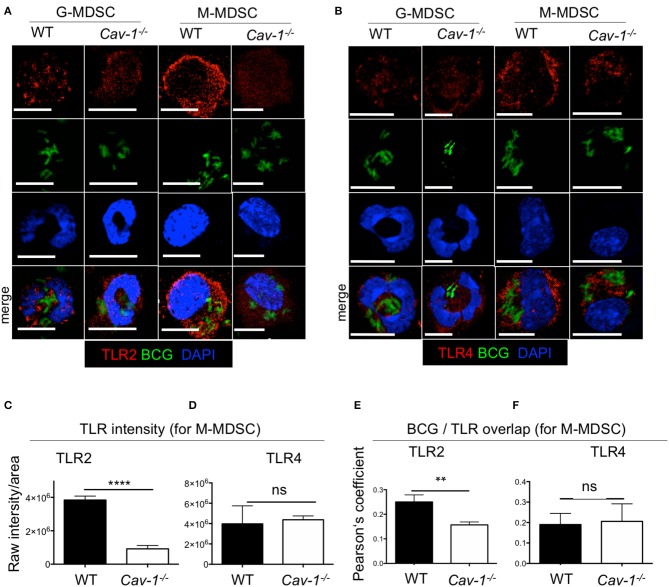
Cav-1 deficient MDSCs show reduced intracellular TLR2 but not TLR4 in BCG-infected vesicles. MDSCs were stimulated with BCG-GFP for 16 h at 1 MOI. Cytospins were stained for TLR2 **(A)** or TLR4 **(B)** and analyzed by confocal microscopy. G-MDSCs and M-MDSCs were identified on the basis of polymorphonuclear or mononuclear shape by DAPI staining. All scale bars 11μm. **(C,D)** Quantified data of raw intensity/area for TLR2 or TLR4. of M-MDSCs from WT and *Cav1*^−/−^ mice. **(E,F)** Pearson's correlation coefficients for colocalization of BCG with TLR2 overlap and BCG with TLR4 overlap. Data represent for **(C)** *n* = 30 cells, for **(D)** *n* = 30 cells, and for **(E,F)** *n* = 10 cells. ****P < 0.0001; **P < 0.01; ns, not significant unpaired, two-tailed, student's *t*-test.

**Figure 3:**
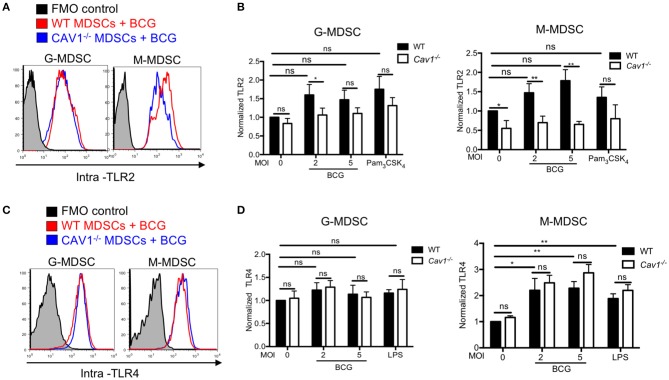
Cav-1 deficient MDSCs have reduced intracellular TLR2 but not TLR4 upon BCG infection. **(A)** MDSCs from WT or *Cav1*^−/−^ mice were stimulated for 16 h with BCG at 2 or 5 MOI and analyzed for intracellular TLR2 expression, or by flow cytometry on G-MDSCs and M-MDSCs subsets using the gates as for Figure 1A. **(B)** Pooled and normalized data of several experiments as performed for **(A)**, and MDSCs were stimulated additionally with the TLR2 agonist Pam_3_CSK_4_. **(C)** Experimental setting as in **(A)**, but MDSC analysis for intracellular TLR4. **(D)** Pooled and normalized data of several experiments as performed for **(C)**, and MDSCs were stimulated additionally with the TLR4 agonist LPS. Normalization of **(B,D)** was done according to unstimulated WT controls. Data are from *n* = 5, independent experiments. **P < 0.01; *P < 0.05; ns, not significant unpaired, two-tailed, student's *t*-test.

Previous reports have shown that the TLR4 recycled between the Golgi apparatus and the cell membrane (33); and in macrophages TLR2 localized around yeast containing phagosomes (34). To address whether intracellular TLR2 and TLR4 are affected by Cav-1, we analyzed both markers intracellularly by confocal microscopy and intracellular FACS analysis using BCG that expressing GFP. Both TLR2 and TLR4 were detected at the cell surface and in the cytoplasm (Figures 2A,B). Surprisingly, we observed that MDSCs from *Cav1*^−/−^ mice had reduced expression of intracellular TLR2, most prominently for M-MDSCs (Figures 2A,C), but not of TLR4 (Figures 2B,D). Moreover, the intracellular colocalization of BCG with TLR2 but not TLR4 was also reduced in MDSCs from *Cav1*^−/−^ mice (Figures 2E,F). This was further confirmed by intracellular staining of TLR2 and TLR4 expression in BCG-infected WT and *Cav-1*^−/−^ MDSCs by flow cytometry. Also, here, TLR2 expression was diminished in unstimulated *Cav-1*^−/−^ in M-MDSCs and the expression was increased by trend but not significantly after BCG infection (Figures 3A,B). In contrast, TLR4 expression was upregulated in M-MDSCs from both WT and *Cav-1*^−/−^ mice upon BCG infection but remained without difference between WT and *Cav1*^−/−^ MDSCs (Figures 3C,D). TLR4 expression remained unchanged by G-MDSC and also showed no effect by Cav-1 deficiency (Figures 3C,D). Together, our results indicate that Cav-1 deficiency affects the intracellular levels of TLR2 that appear as punctual stainings in BCG-infected M-MDSCs, thus suggesting a vesicular localization. Also, BCG-induced up-regulation of intracellular TLR2 is impaired in Cav-1 deficient M-MDSCs.

### Cav-1 Inhibition or Genetic Deficiency Does Not Impair BCG Uptake Into MDSCs and Cav-1 Does Not Co-localize With BCG

**Figure 4:**
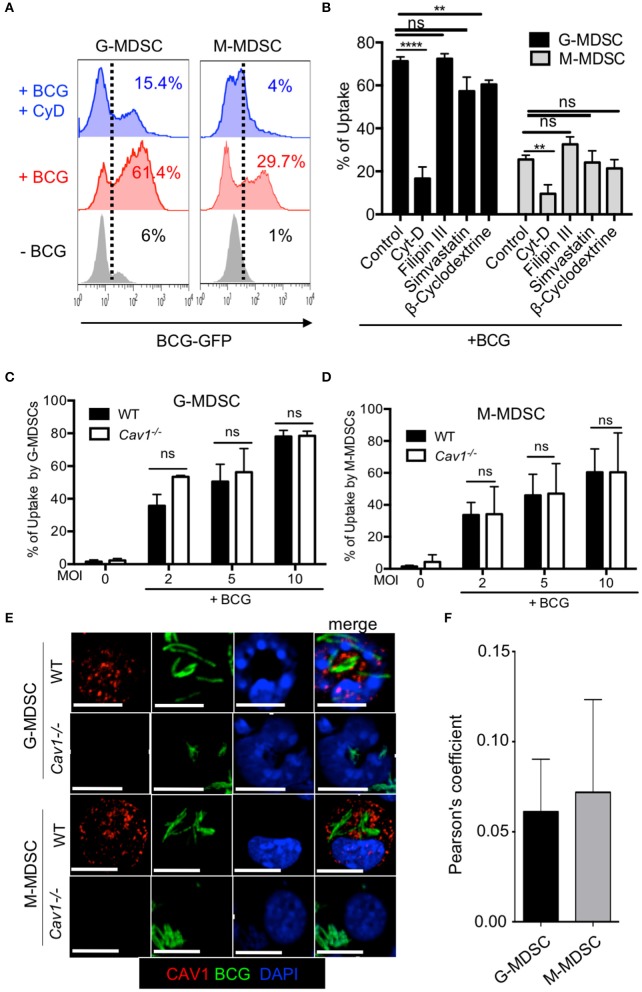
Pharmacological inhibition or genetic deficiency of Cav-1 do not impair BCG uptake by G-MDSC and M-MDSC. **(A)** MDSCs were left untreated or pretreated or not with Cytochalasin D for 1 h and then incubated with BCG-GFP at MOI 2 for 6 h. Cells were then analyzed by flow cytometry for % BCG uptake by GFP detection (gated by dotted line) in G-MDSC and M-MDSC subsets by gates shown in Figure 1A. Dotted lines indicate gating for positive detection of uptake by GFP fluorescence. **(B)** As in A but several pooled experiments are shown and MDSCs were incubated with cytochalasin-D, filipin III, simvastatin or β-cyclodextrine for 1 h and then stimulated with BCG-GFP at MOI of 2, 5, or 10 for 6 h. **(C,D)** MDSCs of WT and *Cav1^−/−^* mice were incubated with BCG-GFP at MOI 2 for 6 h. Cells were then analyzed by flow cytometry for BCG uptake by GFP detection in G-MDSC and M-MDSC subsets by gating as shown in Figure 1A. Pooled and normalized data of several experiments as performed for **(A)**. **(E)** MDSCs were stimulated with BCG-GFP at 1 MOI for 16 h. Cytospins were then stained for Cav-1 and DAPI and analyzed by confocal microcopy. G-MDSC and M-MDSCs were defined on the basis of their nuclear shape. Scale bars upper row 7 μm, all other rows 10 μm. **(F)** Pearson's correlation coefficients for colocalization (“overlap”) of BCG and Cav-1 from *n* = 5 independent experiments like shown in **(E)**. Data are from ****P < 0.0001; **P < 0.01; ns, not significant unpaired, two-tailed, student's *t*-test.

MDSCs have been shown to internalize mycobacteria in infected mice (6) and Cav-1 has been reported to be involved in the uptake of several pathogens (22–24, 35). Therefore, we examined whether Cav-1 is required for BCG uptake into MDSCs. Cytochalasin-D was used as a positive control for blocking actin polymerization required for phagocytosis (36). MDSCs were treated with these inhibitors prior to BCG-GFP infection and then tested for uptake after 6 h by flow cytometry. As expected, cytochalasin D strongly inhibited BCG uptake by phagocytosis (Figure 4A). Filipin III is a cholesterol binding drug which acts as a caveolae disrupter (23, 37, 38). In macrophages, Filipin III has been implicated as functionally important for caveolae-mediated endocytosis (39). Simvastatin and β-cyclodextrine are a lipid raft disrupter drugs which also influence caveolae functions, although less specific (24). Pharmacological inhibition by filipin-III did not block the BCG uptake into G-MDSC or M-MDSC. However, inhibition with β-cyclodextrine reduced the uptake significantly in both G-MDSC and M-MDSC subsets, while simvastatin showed only a trend for reduction of uptake, but without statistical significance (Figure 4B). To further address the role of Cav-1 in BCG uptake, we compared WT with *Cav1*^−/−^ deficient MDSCs. As observed with the pharmacological inhibitors, we did not find any significant difference in the phagocytosis of BCG into WT and *Cav1*^−/−^ G-MDSCs or M-MDSCs (Figures 4C,D).

The formation of caveosomes has been described after mycobacterial uptake into a macrophage cell line J774 (40). Therefore, we tested for the formation of caveosomes in BCG-stimulated MDSCs by confocal microscopy. G-MDSC were identified by their ring-shaped or polymorphic nuclei whereas M-MDSC has kidney shaped or round nuclei. Both MDSC subsets readily ingested BCG-GFP but showed very limited co-localization with Cav-1 (Figure 4E) with a Pearson's coefficient of only +0.05 (Figure 4F). These results show that Cav-1 is dispensable for BCG uptake by MDSCs and do not provide evidence for caveosome formation of BCG in MDSCs.

### Cav-1 Deficiency Alters Inhibitory Markers and Influences Cytokine Production in MDSCs Upon BCG Infection

**Figure 5:**
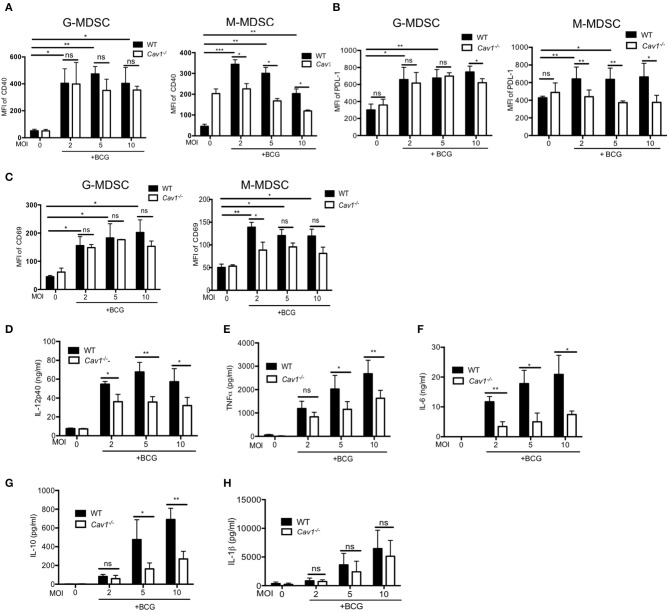
Cav-1 deficiency alters the surface marker profile and impairs the cytokine production after BCG infection selectively of M-MDSCs. MDSCs from WT or *Cav1*^−/−^ mice were stimulated with BCG for 16 h at 2, 5, or 10 MOI. Cells were then harvested and G-MDSCs and M-MDSCs were separately analyzed by flow cytometry for CD40 **(A)**, PD-L1 **(B)**, CD69 **(C)**. Cell supernatants from WT or *Cav1*^−/−^ MDSCs were stimulated with BCG for 16 h at 2, 5, or 10 MOI and were measured by ELISA for the indicated cytokines **(D–H)**. Data shown are from *n* = 3–6 independent experiments. ***P < 0.001; **P < 0.01; *P < 0.05; ns, not significant unpaired, two-tailed, student's *t*-test.

Next, we compared the activation status and inhibitory molecules of WT and *Cav1*^−/−^ MDSCs by flow cytometry. BCG infection resulted in significantly increased CD40, PD-L1, and CD69 expression in G-MDSCs and M-MDSCs from WT mice (Figures 5A–C). However, *Cav1*^−/−^ M-MDSCs had significantly reduced expression of all three markers upon BCG infection while G-MDSCs remained unaffected (Figures 5A–C). We further evaluated the role of Cav-1 in cytokine production in response to BCG infection. We stimulated WT and *Cav1*^−/−^ MDSCs with BCG at increasing MOIs and analyzed for the cytokine production after 16 h by ELISA. Although Cav-1 deficiency affected the secretion of IL-6, IL-12p40, IL-10, and TNF-α in response to BCG infection (Figures 5D–G), no significant difference was observed for IL-1β secretion (Figure 5H). Together, these data suggest that Cav-1 deficiency affects surface markers and secretion of selected cytokines in BCG-infected M-MDSCs but not in G-MDSCs, and that their inflammasome-dependent IL-1β production is Cav-1 independent.

### Lack of Cav-1 Impairs iNOS in BCG-Activated MDSCs and Exhibits Reduced T Cell Suppression

**Figure 6:**
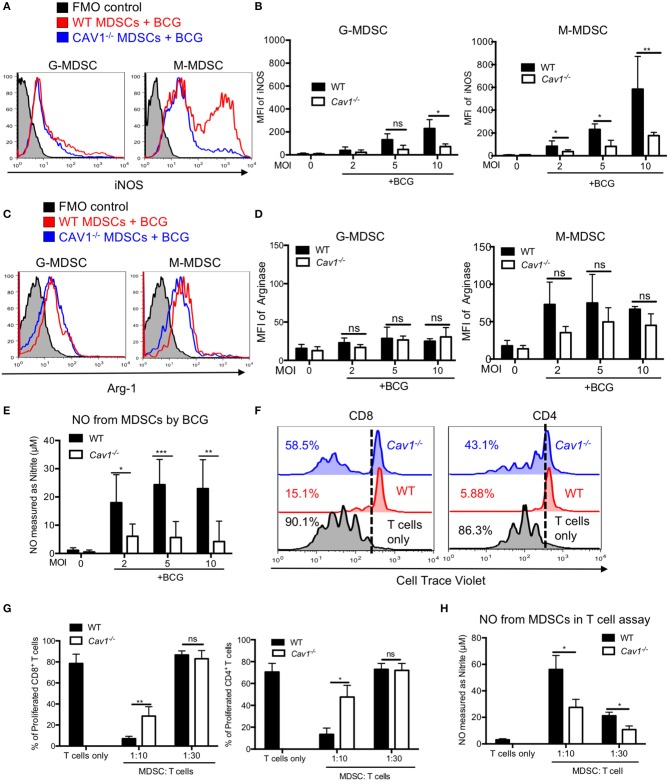
Cav-1 deficiency impairs iNOS expression and reduced T cell suppression by BCG-activated MDSCs. **(A)** MDSCs were stimulated with BCG for 16 h at 2, 5, or 10 MOI. Cells were then harvested and G-MDSCs and M-MDSCs were separately analyzed by flow cytometry for intracellular iNOS. **(B)** Pooled and normalized data of several experiments as performed for **(A)**. **(C)** Experimental setting as in **(A)** but staining for intracellular Arg1. **(D)** Pooled and normalized data of several experiments as performed for **(C)**. **(E)** Cell supernatants from WT and *Cav-1*^−/−^ MDSCs were stimulated with BCG-GFP at 2, 5, or 10 MOI for 16 h. Then NO was measured as nitrite by Griess reaction. **(F)** Suppressor assay for T cell proliferation. Syngeneic lymph node and spleen cells as a source of T cells were labeled with the proliferation dye Cell Trace Violet and then stimulated with anti-CD3 and anti-CD28. Then 1 h BCG pre-activated MDSCs from WT and *Cav-1*^−/−^ mice were added or, as a control, T cells remained without MDSCs. Co-cultures were analyzed after 3 days. Cells were harvested and stained for CD4 and CD8 and analyzed by FACS. T cell division is analyzed as % proliferating cells. Dotted lines indicate the gate separating Cell Trace Violet low proliferating cells from high non-proliferating cells. **(G)** Pooled and normalized data of several experiments as performed for F of proliferated CD8^+^ and CD4^+^ T cells. **(H)** Cell supernatants from the suppressor assay were measured for NO production by Griess assay. Data shown are from *n* = 3–7 independent experiments. ***P < 0.001; **P < 0.01; *P < 0.05; ns, not significant unpaired, two-tailed, student's *t*-test.

L-Arginine degradation by Arg-1 or iNOS, resulting in L-arginine deprivation and NO secretion, respectively, represent major suppressive mechanisms of MDSCs. Hence, we also compared intracellular iNOS and Arg-1 in BCG activated WT and *Cav1*^−/−^ MDSCs by flow cytometry. Interestingly, both G-MDSCs and M-MDSC showed a highly impaired iNOS induction upon BCG infection in the absence of Cav-1 (Figures 6A,B) but no clear difference for Arg-1 (Figures 6C,D). These iNOS data are concordant with the finding that MDSCs from *Cav1*^−/−^ mice also showed massively reduced NO secretion upon BCG infection as compared with WT MDSCs (Figure 6E).

As we observed impaired surface marker and iNOS expression as well as reduced cytokine and NO secretion from *Cav1*^−/−^ MDSCs in response to BCG infection, we hypothesized that MDSCs from *Cav1*^−/−^ mice might also be impaired in their T cell suppression capacity. To test this, we performed an *in vitro* T cell suppression assay where BCG-infected MDSCs were added at different ratios to CD3/CD28 antibody activated T cells to stimulate their proliferation. MDSCs from *Cav1*^−/−^ mice displayed a reduced CD4^+^ and CD8^+^ T cell suppressive capacity when compared to WT MDSCs (Figures 6F,G). Furthermore, supernatants obtained from T cell suppressor assays contained less NO compared to WT MDSCs (Figure 6H). Taken together, these results show that Cav-1 deficiency of MDSCs impairs iNOS expression and thereby NO secretion that was associated with a reduction in T cell suppressive capacity.

### Cav-1 Is Required for p38 MAPK Signaling in BCG-Activated MDSCs

**Figure 7:**
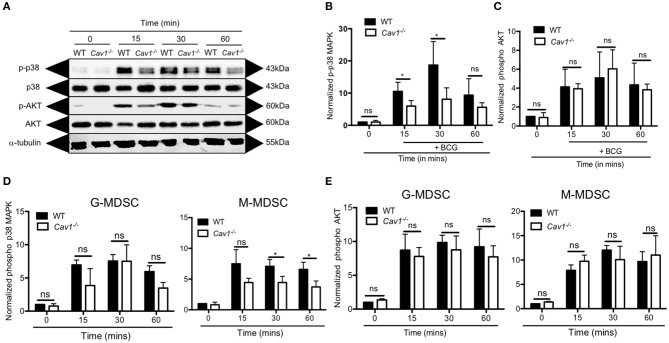
Cav-1 is required for p38-MAPK signaling in MDSCs upon BCG infection. MDSCs from WT or *Cav1*^−/−^ mice were stimulated at 5 MOI BCG for the indicated time periods. **(A)** Cell lysates were prepared, and Western blot analysis was used to examine the total and phosphorylated forms of p38, AKT1 and the housekeeping protein β-tubulin. Normalized and quantified data for phospho-p38 MAP Kinase **(B)** or phospho-AKT **(C)**. **(D,E)** Experimental setting as in **(A)** but G-MDSCs and M-MDSCs were analyzed separately for phospho-p38 **(D)** or phospho-AKT **(E)** by flow cytometry. Normalization of **(D,E)** was done by comparing them to unstimulated WT controls at 0 min. Data shown are from *n* = 3–5 independent experiments. *P < 0.05; ns, not significant unpaired, two-tailed, student's *t*-test.

Next, we addressed the signaling pathway leading to Cav-1 mediated reduction of NO production and thereby decreased T cell suppression. Recognition of mycobacterial ligand by TLR2 can activate MAPK p38 or AKT which are required for NO secretion. For that, we stimulated MDSCs from WT and *Cav1*^−/−^ mice with BCG at indicated time points and analyzed by western blot for the native and phosphorylated forms of p38 and AKT. We found that *Cav1*^−/−^ MDSCs generated less p38 MAPK compared to WT MDSCs (Figures 7A,B and Supplementary Figure 2). However, there was no statistically significant difference observed in p-AKT of WT and Cav-1^−/−^ MDSCs (Figures 7A,C). We also confirmed and extended the p38 and AKT data by flow cytometry showing that specifically the subset of M-MDSCs had reduced p38 MAPK production in the absence of Cav-1 as compared to WT M-MDSCs (Figures 7D,E). No significant difference was observed in G-MDSCs (Figures 7D,E). These results indicate that Cav-1 is required to coordinate BCG activation of TLR2 mediated signals via p38-MAP Kinase in M-MDSCs.

## Discussion

MDSCs are significantly upregulated in both TB patients and murine Mtb infection (4, 6). Here, we provide insights into the interaction of MDSCs with mycobacteria. Both TLR2 and TLR4 are major receptors for mycobacterial recognition (41) but differences in their regulation and signaling in MDSCs have not been elucidated. Our study unveils a role of Cav-1 for mycobacterial recognition and the induction of MDSC functions. We found that Cav-1 affects specifically TLR2 via the p38 MAPK, but TLR4 signaling and the AKT pathway were not affected. This resulted in an impaired induction of surface markers, secretion of cytokines and NO production, and defective T cell suppression. Thereby, MDSCs respond different to mycobacteria as compared to macrophages and DCs. These data extend our general understanding of mycobacterial recognition by immune cells and pathology of TB and may help to develop new treatments for TB by targeting MDSCs (42).

Cav-1 is one of main components of lipid raft invaginations of the plasma membrane expressed on almost all immune cell types (16, 17, 43, 44). In this study, we showed that Cav-1 is upregulated in both G-MDSC and M-MDSCs in BCG infected murine G-MDSC and M-MDSCs. Our data add on previous findings showing Cav-1 upregulation in HIV infected macrophages (32). We detected some differences between G-MDSDs and M-MDSCs. M-MDSCs showed no up-regulation of TLR4 on the cell surface after BCG stimulation, as compared to TLR2. Also the intracellular levels of TLR2 appear more affected in M-MDSC. G-MDSCs showed an up-regulation of CD40. CD69 and PD-L1, on both WT and Cav1-deficient cells. This was in contrast to M-MDSCs, that showed a clear defect when Cav1-deficient, a likely result of their impaired signaling through the p38 MAPK and potentially other, not investigated pathways. Since M-MDSCs are also the major producers of NO via iNOS activity, this subset seems be preferentially affected by Cav1-deficiency. Thus, our data indicate a TLR2-p38-iNOS signaling cascade in M-MDSCs that is Cav-1 dependent and required for T cell suppression.

Cav-1 mediated endocytosis in dendritic cells, macrophages, neutrophils and kidney fibroblast cells has been implicated for several pathogens such as, respiratory syncia virus, *Leishmania chagasi, Pseudomonas aeruginosa, E. coli*, SV40 (simian virus) (21, 23, 45). Surprisingly, we did not find any differences between WT or *Cav1*^−/−^ in the phagocytosis of BCG into G-MDSC or M-MDSCs. Previous reports showed a role of Cav-1 for pathogen entry by using pharmacological inhibitors to block caveolae (45, 46). Others have shown the role of Cav-1 in phagocytosis by using *Cav1*^−/−^ mice for pathogens such as *Pseudomonas aeruginosa and E. coli* (28, 47). In our experimental set up, both pharmacological inhibitors and genetic deficiency of Cav-1 did not show any influence on BCG phagocytosis into MDSCs. After uptake into a macrophage cell line the accumulation of Mtb in caveosomes has been reported (48). Since coronin-1 inhibits the fusion of cytoplasmic vesicles with lysosomes for bacterial degradation (40), this may reflect a mechanism of immune evasion. We readily observed focal, caveolae-like antibody staining for Cav-1 on *Cav1*^−/−^ cells, suggesting cross-reactivity with Cav-2, but at lower affinity. Cav-2 is expressed in macrophages can form heterooligomers with Cav-1 (49). Careful titrations of the antibody allowed to eliminate this background, which was possible by the use of *Cav1*^−/−^ cells. After this, we did not find evidence for a co-localization of Cav-1 with genetically GFP-labeled BCG within G-MDSCs or M-MDSCs. Together, these results indicate that caveosome formation by BCG is either different between subsets of immunogenic (macrophages and DCs) and suppressive immune cells (MDSCs), or Cav-2 detection may account for previous findings and that Cav-2 but not Cav-1 may associate with mycobacteria-containing phagosomes.

We found that CD40, CD69, and PD-L1 surface markers were not up-regulated after BCG infection of *Cav1*^−/−^ MDSCs. In dendritic cells, CD40 ligation results in the recruitment of TRAF6 and further activating p38 MAPK which results in the secretion of cytokines (50). CD40 expression on MDSCs has been shown to be important for suppressive activity and MDSC-mediated Treg expansion in tumor bearing mice (51). In this study, we noted that *Cav1*^−/−^ M-MDSCs dampened intracellular TLR2 and CD40 expression upon BCG infection and impaired activation of p38 MAPK, and thereby reduced T cell suppression. We did not test for induction of Treg in the suppressor assay and consider their expansion unlikely in this short-term assay. The expression of the early activation marker CD69 and the T cell inhibitory ligand PD-L1 were not induced to the same levels in *Cav1*^−/−^ M-MDSCs as compared to WT cells. PD-L1 was increased on blood MDSCs of active TB patients compared to healthy controls (52). Although not further tested here, impaired induction of CD69 and PD-L1 may can be considered as reduced MDSC activation with consequences for known inhibitory effects via PD-L1 in other contexts. The inhibition of activation markers was observed together with a reduced cytokine secretion in *Cav1*^−/−^ M-MDSCs. Silencing of Cav-1 by siRNA in murine alveolar and peritoneal macrophages has been shown to result in increased LPS-induced TNFα and IL-6 and decreased IL-10 production (14). Thus, in macrophages and MDSCs Cav-1 seem to have opposite effects on cytokine secretion. These data also suggest that in M-MDSCs both the inhibitory marker expression and cytokine production depend on Cav-1 to mediate TLR2 expression and signaling after BCG infection.

MDSCs can express high amount of both Arg-1 and iNOS which are involved in the suppressor T cell function (53). Deficiency in arginine-1 inhibits T cell proliferation by impairing CD3 ζ-chain synthesis, which as a consequence prevents upregulation of the expression of cell cycle regulators cyclin D3 and cyclin-dependent kinase 4 (54). NO suppresses T cell function by inducing T cell apoptosis or blocking JAK3 and STAT5 function in T cells (53) or by killing DCs (5). We observed that *Cav1*^−/−^ MDSCs displayed defects in the NO production and T cell suppression. Since NO production represents the major mechanism of our MDSCs *in vitro*, these findings may indicate that MDSCs lose their functional property to suppress T cells in the absence of Cav-1.

Lipid rich surfaces can act synergistically with TLRs in enhancing their signaling intensity (55, 56). Cav-1 is typically associated with surface lipid rafts and some reports linked Cav-1 functionally with TLR4 expression and signaling (19, 47). Cav-1 has been shown to be required for the TLR4 expression and signaling in peritoneal macrophages infected (47). In contrast to this, we could not find a differential surface or intracellular expression and up-regulation of TLR4 in the absence of Cav-1 after BCG infection. However, TLR2 showed reduced amounts of TLR2 in the cytoplasm of *Cav1*^−/−^ MDSCs before BCG infection and a failure to increase intracellular TLR2 after infection. Although considered as a surface receptor, TLR2 has been shown recently to perform signaling from intracellular compartments after vaccinia virus or *Lactobacillus* infection or by LTA exposure from *Staphylococcus aureus*. In these studies induction of type-I interferons via IRFs has been identified as the signaling cascade downstream of vesicular TLR2 (57). Here, we find that after phagocytic uptake of BCG the p38 signaling cascade is reduced leading to various defects to up-regulate surface markers, pro-inflammatory cytokines, as well as iNOS to produce NO for T cell suppression. Thus, our data suggest that surface expression and signaling of TLR2 and BCG phagocytosis are not affected by Cav-1, but rather that vesicular TLR2 signaling is strongly affected. This points to a novel vesicular TLR2 signaling pathway, exemplified here for BCG-infected M-MDSCs and that this pathway is regulated by Cav-1.

In MDSCs, the induction of iNOS and NO secretion relies on NF-κB signals induced by TLR stimulation, which need further support by mobilization of the IRF-1 transcription factor (58). The major adapter for TLR signals, MyD88, has been shown to be required for MDSC accumulation in a model of sepsis (59). M-MDSCs accumulated at the BCG infected site requires MyD88-dependent BCG-specific signals to evade the infection site (60). MDSCs can also be activated by IL-1β *in vitro* and *in vivo* through NF-κB pathway (61). These data suggest that NF-κB is also involved in MDSC expansion and immune suppressive function. We also found that *Cav1*^−/−^ MDSCs which failed to up-regulate TLR2 synthesis after BCG infection also showed an impaired p38 MAPK and NF-κB signaling, indicating that these two cooperative pathways act down-stream of TLR2 in M-MDSCs for NO production. Others have shown that *S. aureus* binds both asialoGM1 and TLR2 in lipid rafts leading to synergistic signals in airway epithelial cells (62). The asialoGM1 mediated co-signals have been identified for flagellin binding to TLR5 to enhance NF-κB signals via the ERK pathway (63). These data indicate that different co-receptors or membrane lipid area components may cooperate with specific TLRs to shape specific immune responses. Here, in this study we found cooperation of TLR2 with Cav-1 for mycobacterial recognition.

In conclusion, our data indicate that known recognition of mycobacteria through TLR2 and TLR4 is differentially affected by Cav-1 in murine M-MDSCs. In the absence of Cav-1, the intracellular expression level of TLR2, but not TLR4, and its increase after BCG infection is impaired. Thereby intracellular TLR2 signaling from BCG-containing phagosomes results in a defect of p38 and NF-κB signals affecting several subsequent activation processes such as surface markers, cytokines, iNOS and NO release, finally impairing M-MDSC suppressor function. Thus, we provide novel signaling pathways via intracellular TLR2 induced by BCG. It is tempting to speculate that similar functional consequences occur in human MDSCs such as indicated by up-regulation of Cav-1, TLR2 and TLR4 on MDSCs of TB patients. Further studies are needed to validate this point.

## Data Availability Statement

All datasets generated for this study are included in the article/Supplementary Material.

## Ethics Statement

The animal study was reviewed and approved by Regierung von Unterfranken.

## Author Contributions

VJ, LK, ER, GW, ND, and ML designed the project and interpreted the data. VJ performed the experiments. VJ and ML analyzed the data and prepared the figures. VJ, ND, and ML wrote the manuscript.

### Conflict of Interest

The authors declare that the research was conducted in the absence of any commercial or financial relationships that could be construed as a potential conflict of interest.
