# Supplementary figures and images for: Caveolin-1 Controls Vesicular TLR2 Expression, p38 Signaling and T Cell Suppression in BCG Infected Murine Monocytic Myeloid-Derived Suppressor Cells
Source: Front Immunol. 2019 Dec 3;10:2826. doi: 10.3389/fimmu.2019.02826 (PMC6901706; doi:10.3389/fimmu.2019.02826)

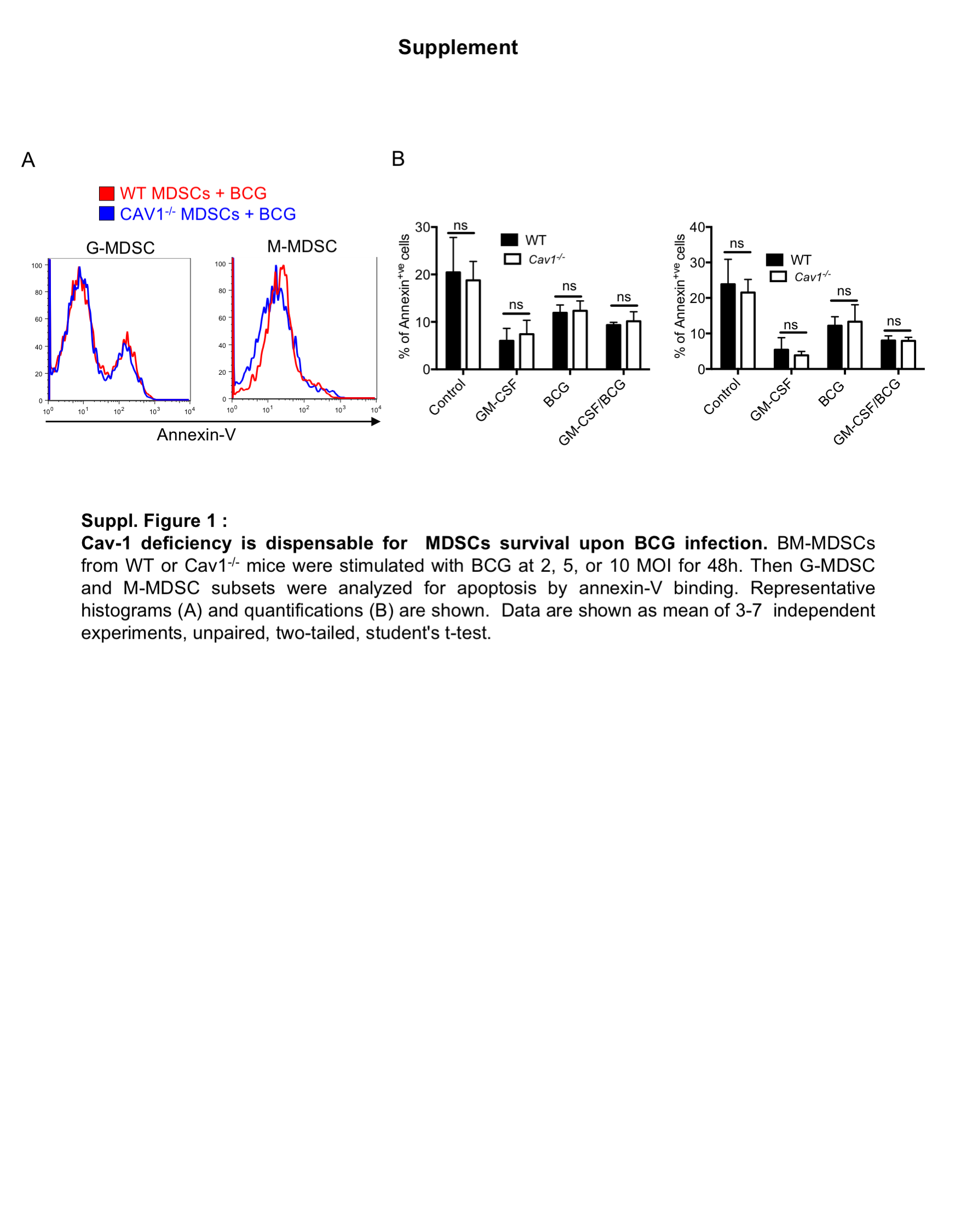

Supplement: Supplementary file 1 [file Image_1.TIFF]

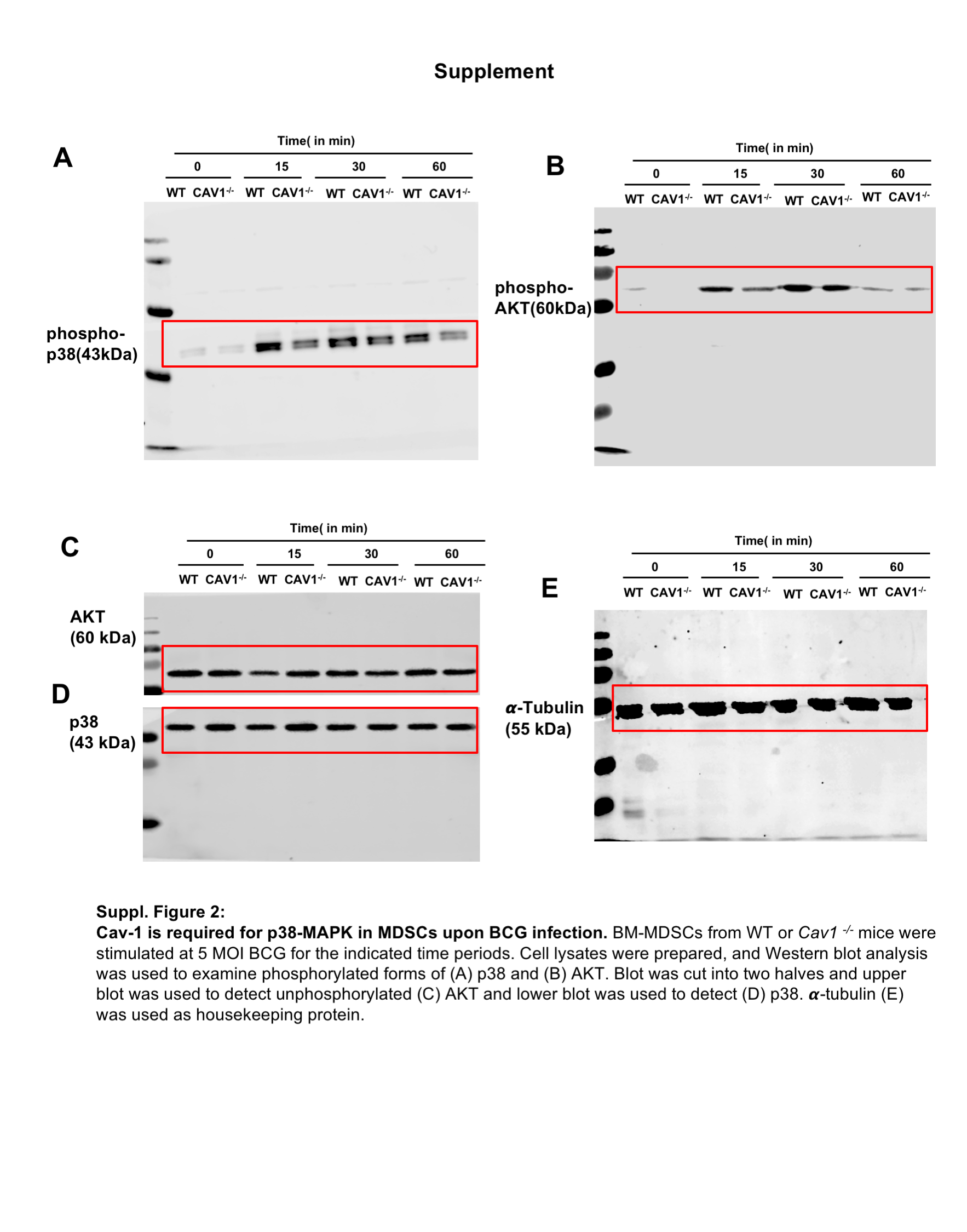

Supplement: Supplementary file 2 [file Image_2.TIFF]
